# Prognostic role of the neutrophil-to-lymphocyte ratio in patients with primary central nervous system lymphoma

**DOI:** 10.18632/oncotarget.20480

**Published:** 2017-08-24

**Authors:** Jongheon Jung, Hyewon Lee, Tak Yun, Eunyoung Lee, Hae Moon, Jungnam Joo, Weon Seo Park, Mihong Choi, Jeong-Ok Lee, Jong Seok Lee, Hyeon-Seok Eom

**Affiliations:** ^1^ Department of Internal Medicine, National Cancer Center, Goyang, Korea; ^2^ Department of Cancer Biomedical Science, Graduate School of Cancer Science and Policy, National Cancer Center, Goyang, Korea; ^3^ Center for Hematologic Malignancy, National Cancer Center, Goyang, Korea; ^4^ Rare Cancers Clinic, Center for Specific Organs Cancer, National Cancer Center, Goyang, Korea; ^5^ Biometrics Research Branch, Research Institute, National Cancer Center, Goyang, Korea; ^6^ Department of Pathology, National Cancer Center, Goyang, Korea; ^7^ Division of Hematology and Medical Oncology, Department of Internal Medicine, Seoul National University Hospital, Seoul, Korea; ^8^ Division of Hematology and Medical Oncology, Department of Internal Medicine, Seoul National University Bundang Hospital, Seoul National University College of Medicine, Seoul, Korea

**Keywords:** primary central nervous system lymphoma, neutrophil-to-lymphocyte ratio, prognosis

## Abstract

Neutrophil-to-lymphocyte ratio (NLR) is one of the parameters of a complete blood cell count (CBC) test and has been reported to be an easily accessible prognostic marker in aggressive cancer, including non-Hodgkin lymphoma (NHL). Primary central nervous system lymphoma (PCNSL) is an extranodal NHL with highly aggressive features. However, the importance of the NLR has never been assessed in PCNSL. This retrospective study enrolled 62 biopsy-proven patients whose baseline NLR was available, and reviewed their medical records to compare both high (≥2.0) and low NLR (<2.0) groups, in terms of clinical characteristics and outcomes. The low NLR group showed significantly better response rates to induction chemotherapy compared to the high NLR group (p=0.041). At a median follow-up of 41.5 months, the high NLR group revealed a significantly worse 3-year overall survival (OS) (42.5 vs. 71.2%; p=0.031) and a worse 3-year progression-free survival (PFS) (37.3 vs. 60.1%; p=0.028). Univariable Cox analysis results showed that a high NLR at diagnosis was a poor prognostic factor for both 3-year OS (HR 2.64, 95% CI 1.06-6.60; p=0.038) and 3-year PFS (HR 2.41, 95% CI 1.07-5.42; p=0.034). However, multivariable analyses adjusting for International Extranodal Lymphoma Study Group (IELSG) score and induction chemotherapy regimen with rituximab, which were strongly prognostic in this study, showed no statistical significance even with the high NLR group’s tendency towards a worse 3-year OS (HR 2.36, 95% CI 0.84-6.62, p=0.102) and a worse 3-year PFS (HR 2.28, 95% CI 0.93-5.63, p=0.073). In conclusion, given that NLR is simple and easily obtainable, it might play a potentially prognostic role in PCNSL from early disease onset.

## INTRODUCTION

Primary central nervous system lymphoma (PCNSL) is a variant of extranodal non-Hodgkin lymphoma (NHL) with highly aggressive features. The survival rate of PCNSL has been improved with high dose methotrexate-based induction chemotherapy. However, its median survival is known to be limited to around 42 months, even with chemotherapy and/or whole-brain radiation [1].

The International Extranodal Lymphoma Study Group (IELSG) presented a prognostic scoring system in 2003, which included risk factors, such as age (>60 years), Eastern Cooperative Oncology Group (ECOG) performance status (>1), elevated serum level of lactate dehydrogenase (LDH), elevated cerebrospinal fluid (CSF) protein concentration and involvement of deep brain regions, *i.e.* periventricular regions, basal ganglia, brainstem and/or cerebellum. The scoring system delineated three strata in terms of prognosis: zero to one, two to three, and four to five risk factors, with a two-year overall survival rate of 80%, 48% and 15%, respectively [2].

Recently, a number of studies have suggested a potential role for neutrophil-to-lymphocyte ratio (NLR), platelet-to-lymphocyte ratio (PLR), red cell distribution width (RDW) and C-reactive protein (CRP) as simple prognostic markers for a variety of cancers [3–8]. Additionally, NLR has been suggested as a pre-treatment prognostic marker, especially for aggressive NHL such as diffuse large B cell lymphoma (DLBCL) and peripheral T-cell lymphoma (PTCL). Troppan et al (2014) demonstrated a value of derived NLR as an independent prognostic factor in DLBCL [9]. Keam et al (2015) also indicated that a high pre-NLR was significantly associated with poor progression-free survival (PFS) and overall survival (OS) of DLBCL treated with R-CHOP [10]. Cengiz et al (2017) validated that a high NLR at diagnosis of mycosis fungoides was positively correlated with both advanced disease stage and disease progression [11]. These results can imply that systemic inflammation is closely related to cancer progression and, from this perspective, systemic inflammatory markers such as NLR, PLR, RDW and CRP might be expected to play an important role in predicting response and survival rates in cancer patients. In addition, these inflammatory markers are not associated with high cost, are easily accessible, and are performed routinely in day-to-day practice. However, these markers have not been explored in PCNSL patients. Thus, our study was aimed to identify the possible prognostic role of NLR and other inflammatory markers in PCNSL patients.

## RESULTS

### Patient characteristics

A total of 62 patients with histologically confirmed PCNSL, diagnosed and treated at the National Cancer Center, Korea and Seoul National University Bundang Hospital between 2001 and 2015, were included in this study. All the histologic results confirmed B-cell lineage lymphoma, with 52 patients diagnosed by stereotactic biopsy and 10 patients diagnosed by surgical resection. All patients received at least one cycle of high dose methotrexate-based induction chemotherapy including methotrexate, vincristine, and procarbazine (MOP), rituximab-MOP (R-MOP), and high-dose methotrexate (HD-MTX). The median age of patients at the time of diagnosis was 62.5 years (range, 21-81), and males comprised 53.2% of the patient group. Baseline characteristics are shown in Table 1. A total of 39 patients (62.9%) were classified into a high NLR (≥2.0) group and 23 patients (37.1%) into a low NLR (<2.0) group. Twenty (32.3%) patients showed ECOG performance status ≥2, which included 17 (43.6%) patients from the high NLR group and three (13.0%) patients from the low NLR group. Comorbidities, including diabetes mellitus, hypertension, cardiovascular diseases, malignancies other than PCNSL, chronic liver disease, and chronic pulmonary diseases, were not significantly different between the two groups. In terms of the IELSG score, 17 (43.6%) in the high NLR group presented a score greater than two, compared to 7 (30.4%) in the low NLR group.

**Table 1:** Clinical characteristics of PCNSL patients

	Total	High NLR (≥2)	Low NLR (<2)	P*
**Number**	62	39 (62.9%)	23 (37.1%)	
**Age (years)**	62.5 (21-81)	61 (21-81)	63 (26-75)	0.805
**>60**	36 (58.1%)	20 (51.3%)	16 (69.6%)	0.253
**≤60**	26 (41.9%)	19 (48.7%)	7 (30.4%)	
**Sex (male)**	33 (53.2%)	22 (56.4%)	11 (47.8%)	0.696
**(female)**	29 (46.8%)	17 (43.6%)	12 (52.2%)	
**ECOG PS**				***0.028***
**0-1**	42 (67.7%)	22 (56.4%)	20 (87.0%)	
**>1**	20 (32.3%)	17 (43.6%)	3 (13.0%)	
**No. of comorbidities**				0.513
**0-1**	47 (75.8%)	28 (71.8%)	19 (82.6%)	
**≥2**	15 (24.2%)	11 (28.2%)	4 (17.4%)	
**IELSG**^†^ **score**				0.225^**^
**0-2**	26 (41.9%)	13 (33.3%)	13 (56.5%)	
**3-5**	24 (38.7%)	17 (43.6%)	7 (30.4%)	
**unknown**	12 (19.4%)	9 (23.1%)	3 (13.1%)	
**Location**				1.000
**non-deep lesion**	34 (54.8%)	21 (53.8%)	13 (56.5%)	
**deep**^‡^ **lesion**	28 (45.2%)	18 (46.2%)	10 (43.5%)	
**CSF cytology**				1.000^††^
**negative**	26 (41.9%)	15 (38.4%)	11 (47.8%)	
**positive**	10 (16.2%)	6 (15.4%)	4 (17.4%)	
**not performed**	26 (41.9%)	18 (46.2%)	8 (34.8%)	
**CSF protein**				0.369^‡‡^
**Normal (<45 mg/dL)**	5 (8.1%)	2 (5.1%)	3 (13.0%)	
**elevated**	30 (48.4%)	19 (48.7%)	11 (47.8%)	
**not performed**	27 (43.5%)	18 (46.2%)	9 (39.1%)	
**LDH**				***0.029***^‡‡^
**normal**	33 (53.2%)	15 (38.4%)	18 (78.3%)	
**elevated**	23 (37.1%)	18 (46.2%)	5 (21.7%)	
**unknown**	6 (15.4%)	6 (15.4%)	0	
**Induction regimen**				1.000^§§^
**MOP^§^**	48 (77.4%)	30 (76.9%)	18 (78.3%)	
**R-MOP║**	13 (21.0%)	8 (20.5%)	5 (21.7%)	
**HD-MTX¶**	1 (1.6%)	1 (2.6%)	0	
**Initial response to induction therapy**				***0.041***^║║^
**CR**	27 (43.5%)	12 (30.8%)	15 (65.2%)	
**CRu**	6 (9.7%)	5 (12.8%)	1 (4.3%)	
**PR**	22 (35.5%)	15 (38.5%)	7 (30.4%)	
**SD**	0	0	0	
**PD**	3 (4.8%)	3 (10.3%)	0	
**Not available**	4 (6.5%)	4 (10.3%)	0	
**WBRT**				0.639
**not performed**	34 (54.8%)	20 (51.3%)	14 (60.9%)	
**performed**	28 (45.2%)	19 (48.7%)	9 (39.1%)	
**Consolidation regimen**				1.000^¶¶^
**VIA^#^**	14 (22.6%)	9 (23.1%)	5 (21.7%)	1.000^##^
**Cytarabine**	20 (32.3%)	13 (33.3%)	7 (30.4%)	
**Other**	2 (3.2%)	1 (2.6%)	1 (4.3%)	
**Not performed**	26 (41.9%)	16 (41.0%)	10 (43.5%)	
**WBC, x10^3^/µL**	6.82 (1.24∼22.6)	7.18 (1.24∼22.6)	5.3 (3.58∼10.53)	***0.001***
**Platelet, x10^3^/µL**	218.15 (63.0)	229.5 (8.68)	199.0 (15.21)	0.065
**Monocyte,/µL**	477.53(212.98)	495.0 (218.12)	447.9 (205.27)	0.405
**ANC, x10^3^/µL**	4.24 (1.57∼20.95)	5.49 (2.32∼20.95)	2.93 (1.57∼5.14)	***<0.001***
**Lymphocyte, x10^3^/µL**	1.66 (0.17∼4.59)	1.52 (0.17∼2.96)	1.89 (1.03∼4.59)	***0.0004***
**NLR**	2.52 (0.83-17.60)	3.58 (2.00-17.6)	1.35 (0.83-1.99)	***<0.001***
**RDW, fl**	13.0(11.4∼19.6)	13.0 (11.4∼19.6)	12.8 (11.6∼14.3)	0.056
**<14.2**	56 (90.3%)	34 (87.2%)	22 (95.7%)	0.398
**≥14.2**	6 (9.7%)	5 (12.8%)	1 (4.3%)	
**PLR**	122.4(53.4∼1626.5)	138.4(77.7∼1626.5)	87.3(53.4∼181.4)	***<0.001***
**<97**	18 (29.0%)	4 (10.3%)	14 (60.9%)	***<0.001***
**≥97**	44 (80.0%)	35 (89.7%)	9 (39.1%)	
**C-reactive protein**				0.516
**Normal**	34 (54.2%)	20 (51.3%)	14 (60.9%)	
**Elevated**	15 (24.2%)	11 (28.2%)	4 (17.4%)	
**not performed**	13 (21.0%)	8 (20.5%)	5 (21.7%)	

RDW, red cell distribution width; PLR, platelet-to-lymphocyte ratio; LDH, lactate dehydrogenase. *Comparison between high-NLR group and low-NLR group, ^†^International Extranodal Lymphoma Study Group, ‡periventricular, basal ganglia, brainstem and/or cerebellum, §methotrexate+vincristine+procarbazine, ║rituximab+MOP, ¶high-dose methotrexate, #etoposide+ifosfamide+cytarabine,**0∼2 vs. 3∼5, † †negative vs. positive, ‡‡normal vs. elevated, §§MOP vs. R-MOP, ║║CR vs. CRu/PR/SD/PD, ¶¶performed vs. not performed, ##VIA vs. cytarabine.

The proportion of elevated LDH was significantly greater in the high NLR group (46.2 vs. 21.7%, p=0.029). Analyses revealed significant differences in white blood cell count (p=0.001), absolute neutrophil count (p<0.001), and lymphocyte count (p=0.0004) between the two groups. The PLR also showed a significant difference between the high and low NLR groups (mean 138.4 vs. 87.3, p<0.001); however, CRP and RDW did not show any meaningful differences.

As for treatments, the two groups showed no significant differences in induction regimens, *i.e.* MOP, R-MOP and HD-MTX. The consolidation regimen distribution and whole brain radiation therapy (WBRT) did not differ between these two groups. By contrast, response rates to induction therapy were significantly different between high and low NLR groups (CR rates, 30.8% vs. 65.2%, respectively; p=0.041).

**Figure 1:**
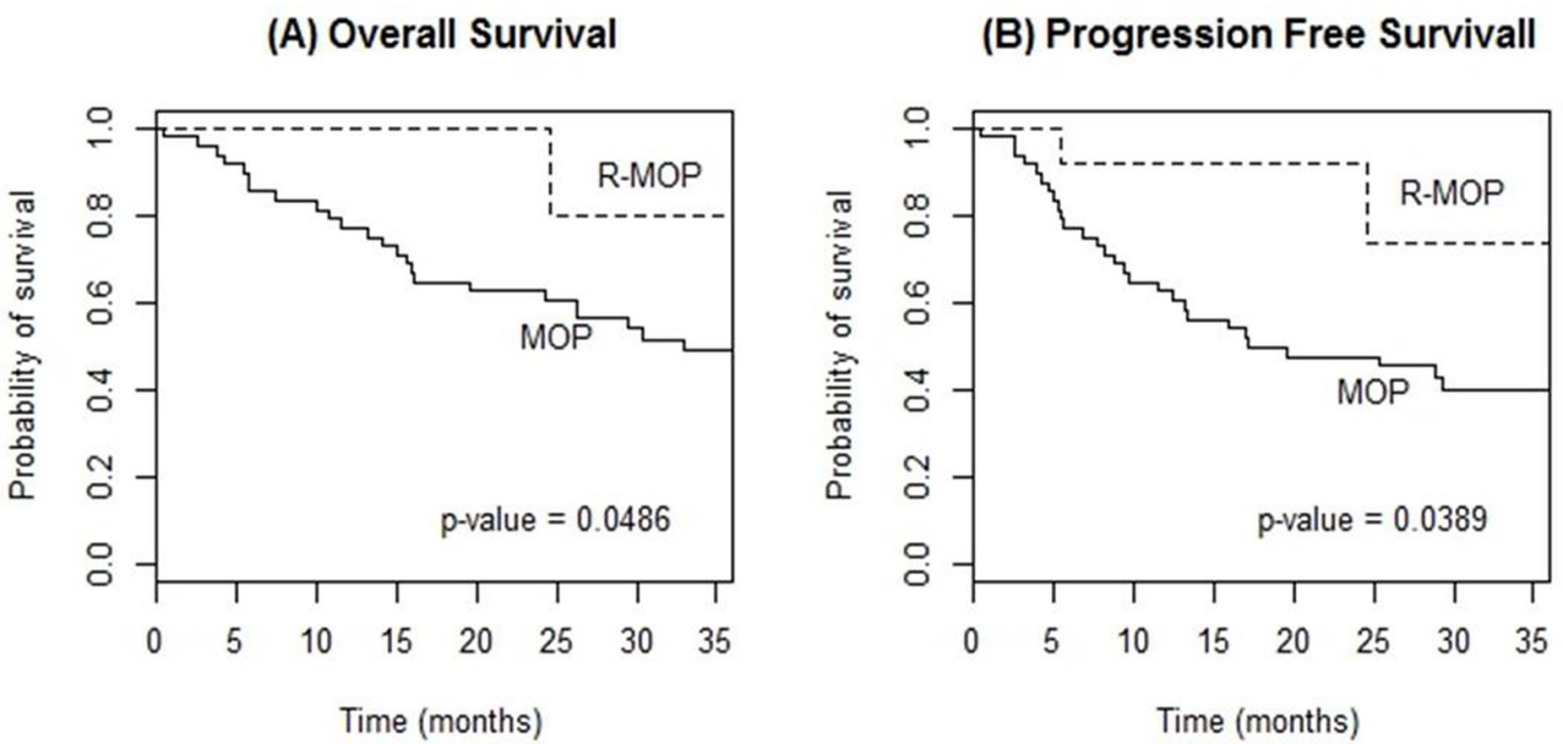
Kaplan-Meier curves of overall survival **(A)** and progression-free survival **(B)** according to induction chemotherapy regimen (R-MOP vs. MOP) in patients with PCNSL.

With a median follow-up of 41.5 (range, 1-124) months, 3-year OS and PFS rates were 50.0% and 41.9%, respectively. Analyses on induction regimens, excluding only one patient who had been treated with HD-MTX, showed a statistically significant difference in 3-year OS (80.0%, R-MOP vs. 49.0%, MOP; p=0.049) and PFS (73.3%, R-MOP vs. 40.0%, MOP; p=0.039) (Figure 1). Regarding consolidation therapies, both WBRT (vs. no WBRT) and etoposide, ifosfamide and cytarabine (VIA) regimen (vs. high-dose cytarabine monotherapy) had a tendency towards a better 3-year OS, albeit not statistically significant (64.5% vs. 57.0%, p=0.363; 67.0% vs. 45.5%, p=0.265, respectively).

During the observation period, 26 deaths occurred. Eight patients died of infectious causes such as septic shock and pneumonia. Four deaths were related to disease progression and others died of unknown causes. Five patients received upfront autologous peripheral blood stem cell transplantation, one of whom died of disease progression. Of the other four patients, one was not followed up and three achieved complete remission and are still being followed up.

### Association between neutrophil-to-lymphocyte ratio (NLR) and clinical outcomes

**Figure 2:**
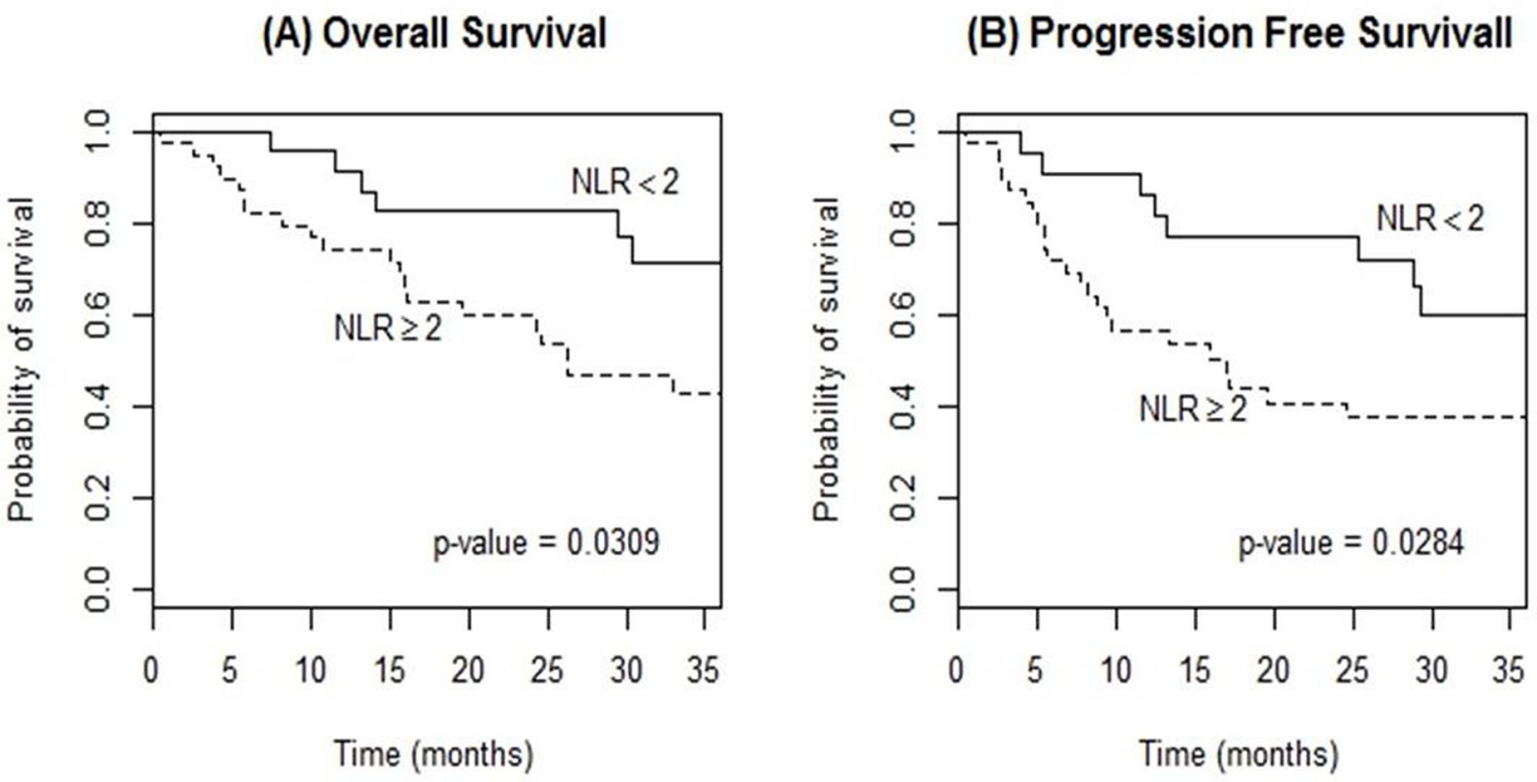
Kaplan-Meier curves of overall survival **(A)** and progression-free survival **(B)** according to neutrophil-to-lymphocyte ratio (NLR) at diagnosis in patients with PCNSL treated with methotrexate-based induction therapy.

Survival analyses demonstrated that the low NLR group had significantly greater OS and PFS compared with the high NLR group: 3-year OS (71.2% vs. 42.5%, p=0.031); 3-year PFS (60.1% vs. 37.3%, p=0.028); respectively (Figure 2). Univariable analyses identified potential prognostic factors affecting OS: NLR ≥2.0 (HR 2.64, 95% CI 1.06-6.60), ECOG Performance score (HR 2.44, 95% CI 1.12-5.34), IELSG score >2 (HR 3.25, 95% CI 1.24-8.51), LDH elevation (HR 2.59, 95% CI 1.15-5.85) and RDW ≥14.2 (HR 3.40, 95% CI 1.26-9.16). Furthermore, NLR ≥2.0, LDH elevation, and RDW ≥14.2 had a statistically significant impact on PFS (HR 2.41, 95% CI 1.07-5.42; HR 2.71, 95% CI 1.28-5.75; and HR 2.71, 95% CI 1.04-7.12, respectively) (Table 2). In multivariable analysis for OS, both the IELSG score >2 and the induction regimen were independent prognostic factors (HR 3.97, 95% CI 1.48-10.7; HR 9.21, 95% CI 1.22-69.8, respectively). NLR at diagnosis was not an independent prognostic factor (HR 2.36, 95% CI 0.84-6.62, p=0.102). In terms of PFS, pre-treatment NLR did not show a significant result (HR 2.28, 95% CI 0.93-5.63, p=0.073) when adjusted with variables similar to the OS model. The IELSG score and the induction regimen also revealed a significant association with PFS (Table 3).

**Table 2:** Univariable analyses for overall survival and progression-free survival

		OS			PFS		
		HR	(95% CI)	*p*	HR	(95% CI)	*p*
NLR	<2	1			1		
	≥2.0	2.64	(1.06, 6.60)	***0.038***	2.41	(1.07, 5.42)	***0.034***
Age (years)	≤60	1			1		
	>60	2.36	(0.99, 5.64)	0.053	2.01	(0.92, 4.36)	0.079
ECOG PS	≤1	1			1		
	>1	2.44	(1.12, 5.34)	***0.025***	1.62	(0.78, 3.34)	0.196
IELSG score	≤2	1			1		
	>2	3.25	(1.24, 8.51)	***0.016***	2.22	(0.97, 5.01)	0.060
Location	non-deep	1			1		
	deep lesion	0.69	(0.31, 1.51)	0.349	0.57	(0.27, 1.20)	0.138
CSF protein	normal	1			1		
	elevated	1.62	(0.20, 12.8)	0.649	2.22	(0.29, 17.1)	0.444
LDH	normal	1			1		
	elevated	2.59	(1.15, 5.85)	***0.022***	2.71	(1.28, 5.75)	***0.009***
Induction regimen	R-MOP	1			1		
	MOP	5.90	(0.80, 43.8)	0.083	4.04	(0.96, 17.0)	0.057
CRP	normal	1			1		
	elevated	0.71	(0.23, 2.17)	0.547	0.95	(0.37, 2.46)	0.921
PLR	<97	1			1		
	≥97	2.82	(0.97, 8.20)	0.057	3.62	(1.26, 10.4)	***0.017***
RDW	<14.2	1			1		
	≥14.2	3.40	(1.26, 9.16)	***0.015***	2.71	(1.04, 7.12)	***0.042***

OS, overall survival; PFS, progression-free survival; HR, hazard ratio; CI, confidence interval; NLR, neutrophil-to-lymphocyte ratio; ECOG, Eastern Cooperative Oncology Group; IELSG, International Extranodal Lymphoma Study Group; CSF, cerebrospinal fluid; WBRT, whole brain radiation therapy; LDH, lactate dehydrogenase; CRP, C-reactive protein; PLR, platelet-to-lymphocyte ration; RDW, red blood cell distribution width.

**Table 3:** Multivariable analyses for overall survival and progression-free survival regarding to pre-treatment NLR

		OS			PFS		
		HR	(95% CI)	*p*	HR	(95% CI)	*p*
NLR	<2.0	1			1		
	≥2.0	2.36	(0.84, 6.62)	0.102	2.28	(0.93, 5.63)	0.073
Induction regimen	R-MOP	1			1		
	MOP	9.21	(1.22, 69.8)	***0.032***	5.51	(1.27, 23.9)	***0.023***
IELSG score	≤2	1			1		
	>2	3.97	(1.48, 10.7)	***0.006***	2.62	(1.12, 6.15)	***0.027***

OS, overall survival; PFS, progression-free survival; HR, hazard ratio; CI, confidence interval; NLR, neutrophil-to-lymphocyte ratio; IELSG, International Extranodal Lymphoma Study Group.

### Association between other biomarkers and clinical outcomes

**Figure 3:**
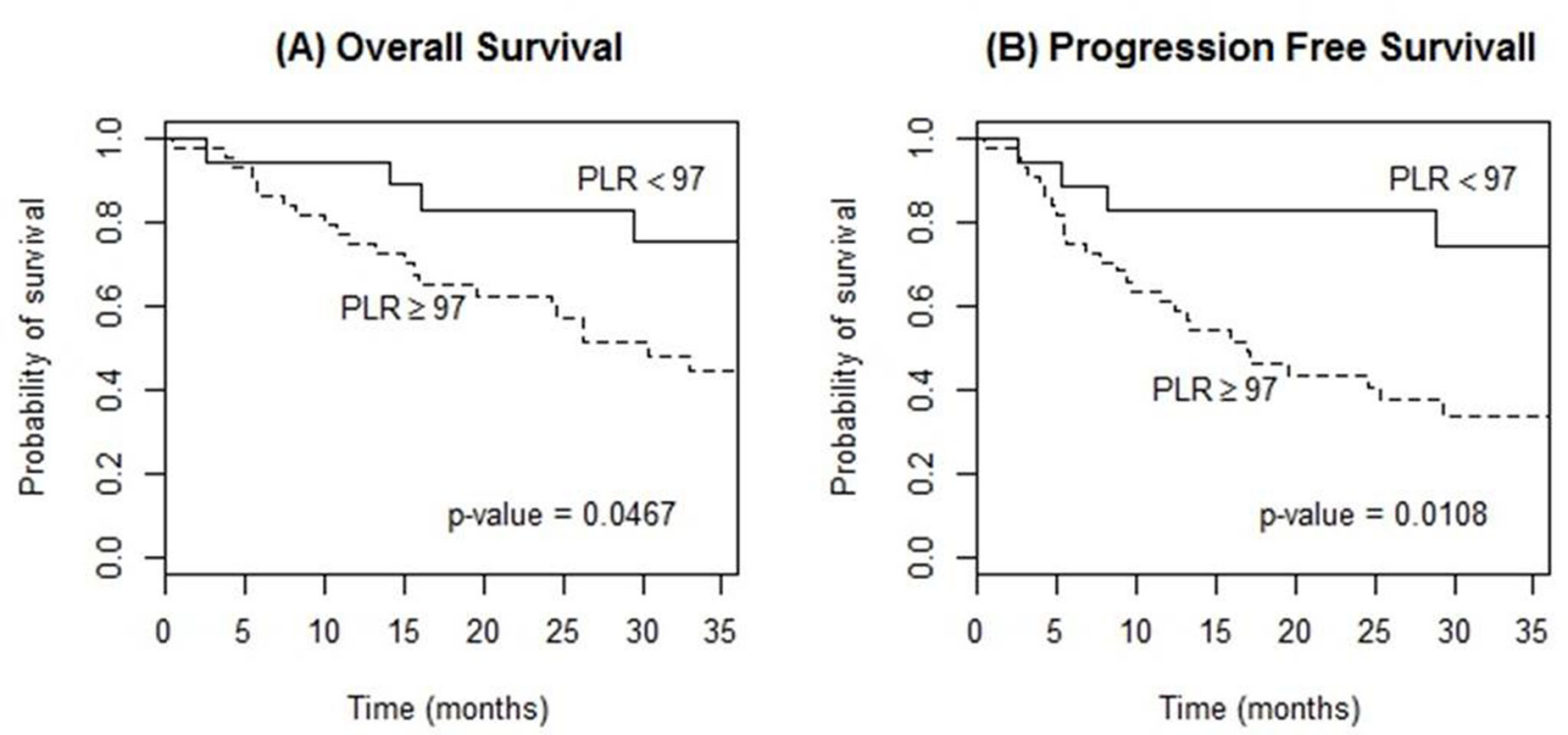
Kaplan-Meier curves of overall survival **(A)** and progression-free survival **(B)** according to platelet-to-lymphocyte ratio (PLR) at diagnosis in patients with PCNSL treated with methotrexate-based induction therapy.

**Figure 4:**
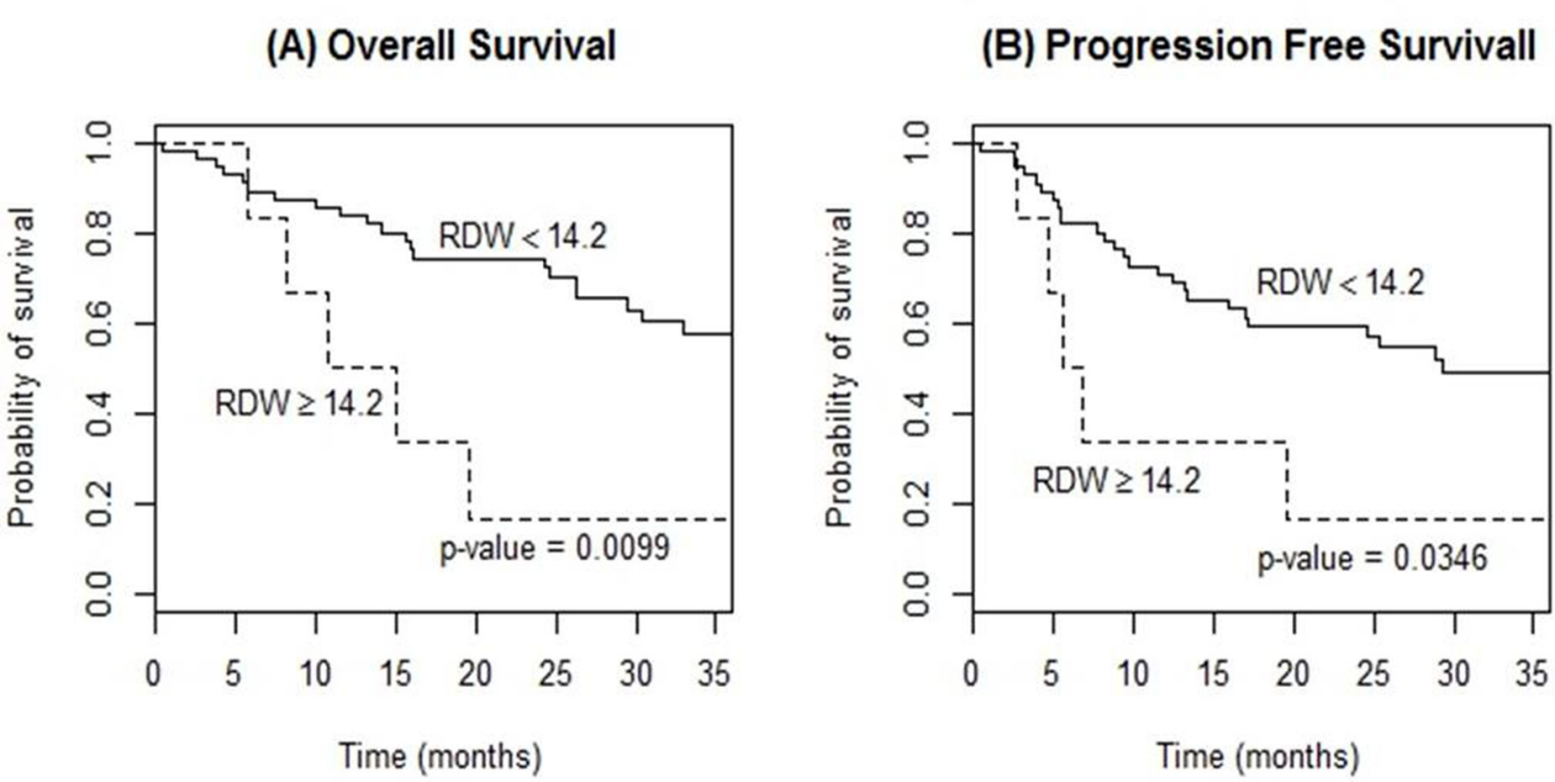
Kaplan-Meier curves of overall survival **(A)** and progression-free survival **(B)** according to red blood cell distribution width (RDW) at diagnosis in patients with PCNSL treated with methotrexate-based induction therapy.

The high PLR group (≥97) showed a significantly poorer OS (3-year OS, 44.4% vs. 75.4%, p=0.047) and PFS (3-year PFS, 33.8% vs. 74.4%, p=0.011) compared to the low PLR group (Figure 3). RDW ≥14.2 also presented a significant relationship for a poorer OS and PFS (3-year OS, 57.5% vs. 16.7%, p=0.010; 3-year PFS, 48.8% vs. 15.2%, p=0.035; respectively) (Figure 4). However, elevated CRP did not reveal any meaningful results. In multivariable analysis, PLR and RDW were not significantly associated with either OS or PFS after adjusting the induction regimen and the IELSG score (Tables 4, 5).

**Table 4:** Multivariable analyses for overall survival and progression-free survival with respect to pre-treatment PLR

		OS			PFS		
		HR	(95% CI)	*p*	HR	(95% CI)	*p*
PLR	<97	1			1		
	≥97	2.24	(0.63, 8.00)	0.214	3.34	(0.95, 11.8)	0.061
Induction regimen	R-MOP	1			1		
	MOP	8.86	(1.17, 67.3)	***0.035***	4.99	(1.15, 21.6)	***0.032***
IELSG score	≤2	1			1		
	>2	3.67	(1.35, 10.0)	***0.011***	2.09	(0.88, 5.00)	0.097

OS, overall survival; PFS, progression-free survival; HR, hazard ratio; CI, confidence interval; PLR, platelet-to-lymphocyte ratio; IELSG, International Extranodal Lymphoma Study Group.

**Table 5:** Multivariable analyses for overall survival and progression-free survival with respect to pre-treatment RDW

		OS			PFS		
		HR	(95% CI)	*p*	HR	(95% CI)	*p*
RDW	<14.2	1			1		
	≥14.2	1.51	(0.43, 5.33)	0.524	1.12	(0.33, 3.88)	0.855
Induction regimen	R-MOP	1			1		
	MOP	8.88	(1.16, 68.2)	***0.036***	4.99	(1.14, 21.9)	***0.033***
IELSG score	≤2	1			1		
	>2	4.37	(1.64, 11.7)	***0.003***	2.85	(1.21, 6.67)	***0.016***

OS, overall survival; PFS, progression-free survival; HR, hazard ratio; CI, confidence interval; RDW; red blood cell distribution width; IELSG, International Extranodal Lymphoma Study Group.

## DISCUSSION

In this study, we performed a retrospective analysis to find biomarkers among clinical variables in patients with PCNSL. First, pre-treatment high NLR showed a possibility of being a poor prognostic marker for a poor OS and PFS. Secondly, pre-treatment high PLR and RDW demonstrated the same poor possibility. Thirdly, we found that induction therapy with R-MOP was significantly associated with better clinical outcomes. Fourthly, we verified that IELSG score was very effective scoring system compared to inflammatory markers, especially in terms of OS. We believe this is the first study to determine the predictive value of pre-treatment NLR in PCNSL patients.

In recent years, many inflammatory markers such as NLR, PLR, RDW, and fibrinogen have been suggested as prognostic markers for various malignancies [3-10, 12-14]. Ho et al. also revealed that the absolute lymphocyte count-to-absolute monocyte count prognostic score (ALC/AMC PS) might provide additional prognostic information to the International Prognostic Index (IPI) for DLBCL [15]. When combined, these results suggest that inflammation and the host immune response interact with each other to develop cancer. Especially with NLR, the neutrophil count represents the innate immune system and affects tumor development. This process is known to be a powerful tumor promoter, producing a conducive environment for tumor growth, facilitating genomic instability, and promoting angiogenesis and lymphangiogenesis by producing chemokines and cytokines [16]. The importance of this process is noteworthy in that many malignancies are initiated by infections – upwards of 15% worldwide [17]. On the other hand, some components of host immunity, such as lymphocytes, represent a beneficial anti-tumor effect [18]. In some previous studies, the relationship between a better clinical outcome and immune cell infiltration has been suggested in melanoma, breast cancer, ovarian cancer, and non-small cell lung cancer [19–23]. In that regard, pre-treatment high NLR could be one of the poor prognostic factors theoretically.

In our study, pre-treatment PLR also showed a possibility of being a prognostic marker for PCNSL. PLR was significantly associated with PFS in a univariable analysis, although we failed to prove a statistically significant relationship between PLR and OS. There have been some suggestions about the role of platelets and the coagulation system in the progression of cancer [24]. The level of circulating platelets correlates with the level of serum vascular endothelial growth factor-A, playing an important role in angiogenesis for tumor progression [25]. In addition, platelets are known to enhance metalloproteinase-9 (MMP-9) secretion, by which platelets promote tumor cell invasion [26]. Also, cancer cells could increase the circulating platelet count by stimulating the proliferation of megakaryocytes and, in this perspective, an increased blood platelet count could be assumed to reflect systemic inflammation induced by cancer [27, 28]. Menter et al. suggested that the interaction between cancer cells and platelets possibly suppresses immune recognition and the elimination of cancer cells [29]. Based on these previous studies, PLR also might be assumed to be a possible prognostic factor. In addition, an elevated pre-treatment RDW presents the possibility of predicting the prognosis. Considering that an elevated RDW has been suggested to reflect tumor burden and inflammatory conditions in previous studies, more research is required to prove a relationship between RDW and the prognosis of PCNSL in the future [8].

The central nervous system normally lacks lymphoid aggregation and it is still unclear whether PCNSL develops locally or systemically [30, 31]. Therefore, one remaining question is how important the systemic inflammatory condition is, represented by NLR, PLR and RDW, in the development of PCNSL. Montesinos-Rongen et al. suggested that the original cell of PCNSL develops outside the CNS and survives in an immunologically aberrant CNS, whereas it should normally have been eradicated by an intact immune system [32]. Considering this possible origin of PCNSL, systemic inflammation still could be assumed to affect the development of PCNSL. In our data, NLR 2.0 was identified as a relatively sensitive and specific point to discriminate high NLR and low NLR groups, but in most previous studies on DLBCL, an NLR of 3.0 to 5.0 was used as the distinguishing point [3, 9, 10, 13, 15]. The relatively low NLR cutoff in our data might reflect that PCNSL could develop in a relatively less severe inflammatory environment or even in an immune-deficient condition. The specificity of CNS is another factor that may contribute to the lower NLR cutoff in our study compared to that used in other studies for extra-CNS lymphoma. The majority of patients with PCNSL succumb to a local relapse or refractoriness rather than distant failure. The CNS, and the brain in particular, is enclosed by skeletal bone where pressure is sensitive to total volume, including water content, and where pressure increases with inflammation. Considering NLR as a surrogate marker for inflammatory burden, we consider it likely that the low NLR cutoff reflects higher vulnerability of PCNSL to inflammation compared to other types of DLBCL, which are mostly systemic by nature [33]. To determine the origin of PCNSL and the most discriminate point of NLR, more studies are required.

In comparison with MOP as an induction chemotherapy, R-MOP showed significantly improved outcomes in our data. The addition of rituximab to conventional chemotherapy has not been firmly validated [34–37]. Although our study included a relatively small number of PCNSL patients (n = 62), our result showed the possible superiority of a rituximab-included regimen as an induction chemotherapy. WBRT is another important therapy for PCNSL [38]. However, no significant difference in clinical outcomes was noticed in the presence or absence of WBRT. Additionally, the study enrolled five patients who had been treated with autologous hematopoietic stem cell transplantation (HSCT). Schorb et al. reported the remarkably high efficacy of high-dose chemotherapy, followed by autologous HSCT, with a response rate of 95% and a median OS rate of approximately ten years [39]. Thus, further investigation is required to determine other possible prognostic factors relating to therapeutic options. Furthermore, more studies are needed to confirm whether efficient therapeutic options such as HSCT could ultimately improve the prognosis of PCNSL.

Our study has several limitations. First, the sample size of 62 was relatively small. However, considering the extreme rarity of PCNSL, the results of our study are worth consideration. A prospective, multi-center study is required for further investigation. Secondly, all the patients in our study presented with histologically confirmed DLBCL. There was one case of T-cell origin PCNSL in our data initially, but this case was excluded in the final analysis due to unavailable pre-treatment laboratory data. However, patients with T-cell origin PCNSL are known to show a similar prognosis to patients with B-cell lineage PCNSL [40]. Therefore, the results may not be biased. Thirdly, some of the previously known risk factors, such as deep location and elevated CSF protein, were not statistically significant. This is possibly due to the small sample size. However, the IELSG score was significantly related to both OS and PFS. Thus, our results still retain their value.

In conclusion, pre-treatment NLR might be a potential prognostic marker for PCNSL. Pre-treatment PLR and RDW also showed the possibility of being prognostic markers and the choice of induction regimen might also be of importance for prognosis. Further studies are needed to verify the additional prognostic values of these factors.

## MATERIALS AND METHODS

### Patients

The investigators identified histologically confirmed PCNSL patients, between 2001 and 2015 at National Cancer Center, Korea and Seoul National University Bundang Hospital, with the following inclusion and exclusion criteria. The inclusion criteria were: 1) aged 18 years or older at diagnosis; 2) having received at least one cycle of methotrexate-based induction therapy and; 3) baseline clinical and laboratory data available from medical records, in particular, prior to initial chemotherapy or glucocorticoid treatment. The exclusion criteria were: 1) radiation therapy in advance of induction chemotherapy; 2) immunodeficiency including human immunodeficiency virus seropositivity and; 3) other malignancies diagnosed during the observation period. Medical records and laboratory results were retrospectively reviewed. This study was approved by the institutional review board of both institutions and conducted in compliance with the Declaration of Helsinki.

### Measurement of laboratory data and response evaluation

Pre-treatment NLR was defined as the value that was obtained on the nearest day within 4 weeks before the initiation of the treatment, including steroid and/or methotrexate-based induction chemotherapy. Laboratory values were measured using XE-2100 (Sysmex, Kobe, Japan) at National Cancer Center and at Seoul National University Bundang Hospital. The NLR was calculated as the absolute neutrophil count measured in x10^9^/L, divided by the absolute lymphocyte count measured in x10^9^/L. The PLR was calculated as the absolute platelet count measured in x10^3^/dL, divided by the absolute lymphocyte count measured in x10^9^/L. We collected data from two institutions in situations where the normal ranges of LDH and CRP differed from each other. We then transformed these values into dichotomous variables, i.e. normal versus elevated. OS was defined as the duration from the diagnosis to death. PFS was measured from the diagnosis to the earliest date that the disease had progressed or to death. The study evaluated the initial responses of induction chemotherapy, using the criteria suggested by the International PCNSL Collaborative Group 2005, which classifies treatment responses into CR (complete response), CRu (CR, unconfirmed), PR (partial response), SD (stable disease) and PD (progressive disease) [41].

### Statistical analysis

Considering that this is the first study about the NLR in the patients with PCNSL and cutoff values of NLR, PLR and RDW for NHLs have been different in every previous study, we decided to apply Contal and O’Quigley’s method using log rank test statistics to find the optimal cutoff point for the time-to-event outcome and determined the cutoff values for PCNSL markers, such as NLR, PLR and RDW [42]. SAS macro %FINDCUT was used to implement the method. Based on pre-treatment NLR levels, patients were divided into a high NLR group (≥2.0) and a low NLR group (<2.0). Patient characteristics and survival outcomes were compared between the two groups. Continuous variables were analyzed using the two-sample t-test or the Wilcoxon rank sum test, depending on the normality test result. Categorical variables were analyzed using χ^2^ test or Fisher’s exact test, as appropriate. For the survival analysis, the Kaplan-Meier method, with a log-rank test, was used. Both univariable and multivariable Cox proportional hazard models were used, adjusting for the influences of potential confounders, to evaluate each biomarker’s prognostic value. Hazard ratios (HRs) estimated from the Cox analysis were presented as relative risks with corresponding 95% confidence intervals (CIs). All statistical analyses were performed using the SAS version 9.4 (SAS Institute Inc., Cary, NC, USA) and R 3.1.2 statistical software. A two-sided *p*<0.05 was considered statistically significant.

## Abbreviations

PCNSL: primary central nervous system lymphoma
NLR: neutrophil-to-lymphocyte ratio
PLR: platelet to lymphocyte ratio
IELSG: International Extranodal Lymphoma Study Group
ECOG: Eastern Cooperative Oncology Group
DLBCL: diffuse large B-cell lymphoma
PTCL: peripheral T-cell lymphoma
HR: hazard ratio
CI: confidence interval
